# A Valid Bisphosphonate Modified Calcium Phosphate-Based Gene Delivery System: Increased Stability and Enhanced Transfection Efficiency In Vitro and In Vivo

**DOI:** 10.3390/pharmaceutics11090468

**Published:** 2019-09-11

**Authors:** Ming Zhao, Ji Li, Dawei Chen, Haiyang Hu

**Affiliations:** School of Pharmacy, Shenyang Pharmaceutical University, No.103, Wenhua Road, Shenyang 110016, China

**Keywords:** gene delivery, CaP nanoparticles, p53 plasmid, bisphosphate, nuclear locating sequences, antitumor activity, folic acid

## Abstract

Calcium phosphate (CaP) nanoparticles, as a promising vehicle for gene delivery, have been widely used owing to their biocompatibility, biodegradability and adsorptive capacity for nucleic acids. Unfortunately, their utility in vivo has been profoundly restricted due to numerous technical barriers such as the lack of tissue specificity and limited transfection efficiency, as well as uncontrollable aggregation over time. To address these issues, an effective conjugate folate-polyethylene glycol-pamidronate (shortened as FA-PEG-Pam) was designed and coated on the surface of CaP/NLS/pDNA (CaP/NDs), forming a versatile gene carrier FA-PEG-Pam/CaP/NDs. Inclusion of FA-PEG-Pam significantly reduced the size of CaP nanoparticles, thus inhibiting the aggregation of CaP nanoparticles. FA-PEG-Pam/CaP/NDs showed better cellular uptake than mPEG-Pam/CaP/NDs, which could be attributed to the high-affinity interactions between FA and highly expressed FR. Meanwhile, FA-PEG-Pam/CaP/NDs had low cytotoxicity and desired effect on inducing apoptosis (71.1%). Furthermore, FA-PEG-Pam/CaP/NDs showed admirable transfection efficiency (63.5%) due to the presence of NLS peptides. What’s more, in vivo studies revealed that the hybrid nanoparticles had supreme antitumor activity (IR% = 58.7%) among the whole preparations. Altogether, FA-PEG-Pam/CaP/NDs was expected to be a hopeful strategy for gene delivery.

## 1. Introduction

Over the past several decades, gene therapy has become one of the most actively developing and promising branches of medicine [1,2,3,4,5]. Calcium phosphate (CaP) nanoparticles, as a promising vehicle for gene delivery, have been widely used because of their biocompatibility, biodegradability and adsorptive capacity for nucleic acids [6,7]. Unfortunately, their utility in vivo has been profoundly restricted due to numerous technical barriers such as the lack of tissue specificity and limited transfection efficiency as well as the uncontrollable growth of CaP crystals in a physiological solution over time [8]. Therefore, how to conquer these obstacles becomes an urgent question in the gene delivery.

To overcome these drawbacks, many methods for stabilizing CaP/DNA particles have been studied. One approach is to increase the concentration of Ca^2+^ ion, which has been demonstrated to retard particle development [9]. Beyond that, the calcium phosphate nanoparticles have been coated with DNA to prevent coagulation [10]. This approach limits the particle growth, but it is relatively intricate and does not apply to gene delivery systems on account of its lack of stealth characteristics. Pamidronate (Pam), as a kind of second generation of organic bisphosphate, is usually employed to treat bone diseases owing to its strong affinity for hydroxyapatite, which is a component of bone tissue and a naturally occurring crystalline form of CaP [11]. Since the so-called “bone hook” hydroxyl group and two phosphonate groups all interact closely with Ca^2+^ ion, the combination of bisphosphates could solve the aggregation of CaP nanoparticles in vitro [12,13,14]. In addition, pamidronate can be easily conjugated with polyethylene glycol (PEG) due to the presence of amino groups. Typically, the attachment of PEG chains is used to form a hydrophilic outer shell for promoting biocompatibility and stability [15].

To further reduce the side effects on normal cells, it is essential to improve tumor cell targeting by attaching the targeting ligands on the surface of nanoparticles. One of the most broadly used small molecules for this is folic acid (FA). Folate receptors (FR) are over-expressed on the surface of numerous tumor cells, yet they are hardly expressed on normal cells [16]. FA has a high binding affinity for FR and nanoparticles conjugated with FA will be subsequently internalized into tumor cells via receptor-mediated endocytosis [17].

Additionally, gene expression of exogenous DNA depends on an essential nuclear importation process. Since free plasmid is restricted out of the nucleus, it needs to be improved in order to utilize nuclear localization sequences to facilitate the active transport of plasmid DNA into the nucleus from the cytoplasm through binding to nuclear transport proteins, thus assuring high gene expression [18]. Simian virus 40 (SV40) large tumor antigen (T-antigen) is a broadly studied and commonly investigated nuclear localization signal (NLS) for the delivery of macromolecules with a well-defined nuclear import pathway [19].

**Figure 1:**
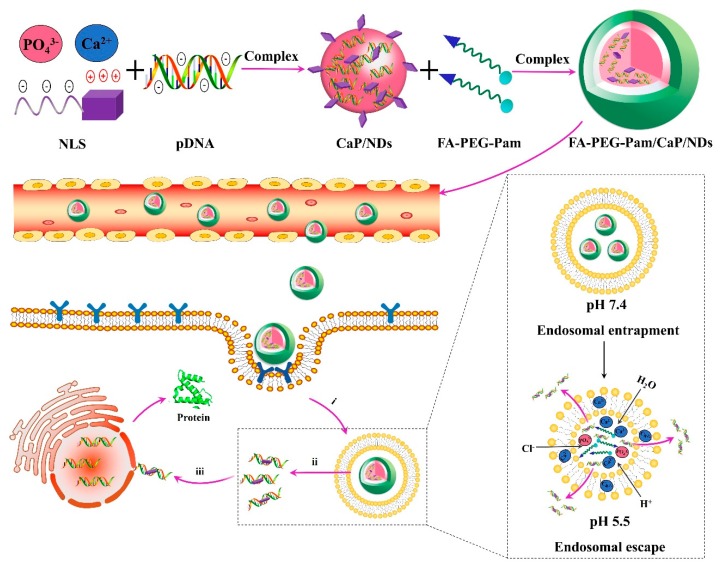
Schematic illustration of the formation of FA-PEG-Pam/CaP/NDs nanoparticles and the extracellular and intracellular trafficking for the systemic delivery of plasmid DNA to tumors. Nanoparticles would be internalized into tumor cells via the FR-mediated endocytosis. After being internalized (i), in the acidic endosomes, FA-PEG-Pam/CaP/NDs nanoparticles would dissolve and the embedded pDNA could escape the endosome by endosome rupture owing to the increase of osmotic pressure (ii). Eventually, pDNA is transported to the nucleus with the aid of NLS due to its nuclear locating ability (iii).

Taking together all these precedents, we develop herein a versatile FA-PEG-Pam/CaP/NDs hybrid nanoparticle (Figure 1). The FA-PEG-Pam shell will help to overcome the physical instability of CaP nanoparticles. When FA-PEG-Pam/CaP/NDs nanoparticles are intravenously administered into the blood system, their stability and circulation time would be increased due to the FA-PEG-Pam shell. Meanwhile, nanoparticles would be internalized into tumor cells via the FR-mediated endocytosis. In acidic endosomes, FA-PEG-Pam/CaP/NDs nanoparticles would dissolve and the embedded pDNA could escape the endosome by endosome rupture owing to the increase of osmotic pressure. Eventually, pDNA is transported to the nucleus with the aid of NLS peptides. This ingenious design may provide effective gene delivery to cancer cells and a high tumor inhibition rates. In addition, p53 plasmid was used as a therapeutic gene to perform the antitumor experiments in vivo.

## 2. Materials and Methods

### 2.1. Materials

Pamidronate (Pam) was bought from Sigma-Aldrich (St. Louis, MO, USA). FA-PEG_2000_-COOH, 1-(3-dimethylaminopropyl)-3-ethylcarbodiimide hydrochloride (EDC) and *N*-hydroxysuccinimide (NHS) were obtained from Aladdin Biochemical Technology Co. Ltd. (Shanghai, China). Lipofectamine 2000 was bought from Invitrogen Life Technologies (Carlsbad, NM, USA). The NLS peptide of the SV40 T antigen (CGGGPKKKRKVED) and a scrambled sequence (NLS(scr), CGGGPKTKRKVED) were synthesized by GenScript Corporation (Shanghai, China). YOYO-1 was bought from Invitrogen (Carlsbad, NM, USA). RPMI-1640 medium and Fetal bovine serum (FBS) were bought from Gibco (New York, NY, USA). Wild p53 (5′-GGCTCTGACTGTACCACCATCCA-3′) was designed from miaoling.bio (Beijing, China) extracted using the EndoFree Plasmid Kit from Qiagen (Frankfurt, Germany). The plasmids pDsRed-M-N1 were purchased from Promega (Madison, Wisconsin, WI, USA). 4T1 cells and HepG2 cells were bought from Nanjing KeyGen Biotech Co. Ltd. (Nanjing, China). Female BALB/c mice (20 ± 2 g) were obtained from Shenyang Changsheng Biotechnology Co. Ltd. (Shenyang, China).

### 2.2. Synthesis of FA-PEG-Pam

The synthesis procedures of nanoparticles is illustrated in Figure S1. Briefly, FA-PEG_2000_-COOH (2.0 g, 1 mmol), EDC (0.23 g, 1.2 mmol), NHS (0.14 g, 1.2 mmol) and triethylamine (0.51 g, 5 mmol) were dissolved in anhydrous dimethyl sulfoxide (DMSO). The mixture was alowed to react for 6 h. Subsequently, pamidronate (0.44 g, 1.2 mmol) was added to the above mixture under stirring and reacted for 24 h. The product was then dialyzed using distilled water for 48 h (MWCO 1 kDa). Finally, FA-PEG-Pam was obtained after lyophilization. The products were characterized by ^1^H-NMR using an AMX 600 MHz spectrometer (Bruker, Karlsruhe, Germany). Meanwhile, freeze-dried polymer samples were pressed with KBr under vacuum and scanned from 4000 to 400 cm^−1^ using a Bruker Equinox 55 FT-IR spectrometer.

### 2.3. Preparation of FA-PEG-Pam/CaP/NDs Nanoparticles

Briefly, 5 uL of pDNA (0.5 ug/μL) was mixed with NLS (SV40) at a weight ratio of 1:1 to form NLS/pDNA (NDs) through electrostatic interaction. After that, 1 mL Ca^2+^ (0.25 M) was added and incubated for 10 min. This Ca^2+^/NDs solution was carefully added to 1 mL phosphate solution (6.0 mM Na_2_HPO_4_, 50 mM HEPES, 300 mM NaCl, pH 7.1) with agitation. Then, 1 mL FA-PEG-Pam solution (10 mg/mL) was mixed with the CaP/NDs solution to form FA-PEG-Pam/CaP/NDs nanoparticles under stirring. Then, the mixture was centrifuged at 8000× *g* for 15 min. Finally, the sediment was resuspended in the 2 mL deionized water. The final concentration of pDNA was 1.25 μg/mL, NLS was 1.25 μg/mL, Ca^2+^ was 27.75 mg/mL and FA-PEG-Pam was 5 mg/mL. Meanwhile, CaP/NDs, mPEG-Pam/CaP/NDs, FA-PEG-Pam/CaP/pDNA and FA-PEG-Pam/CaP/NDs were prepared (The final concentration of CaP and pDNA was the same).

### 2.4. Characterization of the Nanoparticles

The zeta potential and particle size of the nanoparticles were assessed by a Zetasizer Nano ZS (Malvern, UK). The samples were diluted in the deionized water. The measurements were as follows: the laser used in the Malvern Zetasizer was 4 mW HeNe laser of 633 nm wavelength, and it was equipped with avalanche photo diode detectors (APD) with the angel of 173°. The specific experimental parameters included run duration: 10 s; equilibration time: 120 s; and number of runs: 14. The morphology of complexes were scanned through the transmission electron microscopy (TEM, Gatanmodel 794 CCD, bottom mounted, Oxford instruments, Oxford, England). In brief, the samples were placed on the copper mesh. Then, the excessive solution was removed by filter paper and dried at 25 °C.

The encapsulation efficiency (EE%) was determined by the ultrafiltration method [20]. Nanoparticles were added into filter devices (50 kD) and centrifuged at 12,000× *g* for 15 min. Centrifuged solution which contained unbound pDNA were collected. Then solutions were mixed with Hoechst 33258 solution and determined by microplate reader. The fluorescence intensity was assessed at 457 nm (em) and 353 nm (ex). EE% was calculated through the following equation:

EE% = (Ft − Fu)/Ft × 100%
(1)

Fu and Ft were set as the concentration of unloaded and total pDNA, respectively.

### 2.5. In Vitro Release of pDNA

2 mL of freshly prepared FA-PEG-Pam/CaP/NDs nanoparticles solution (1.25 μg/mL pDNA, 27.75 mg/mL Ca^2+^, 5 mg/mL FA-PEG-Pam) was centrifuged at 12,000× *g* for 15 min. Then, the supernatant was removed and the precipitate was suspended in two aqueous buffer solutions (pH 5.5 and pH 7.4). The Hoechst 33258 intercalation assay was carried out to calculate the amount of pDNA released at different time point. Meanwhile, agarose gel electrophoresis on 0.7% agarose gel was used for analysis of pDNA release.

### 2.6. Serum Stability

pDNA, NDs, CaP/NDs, mPEG-Pam/CaP/NDs, FA-PEG-Pam/CaP/pDNA and FA-PEG-Pam/CaP/NDs solution were gently mixed with proper FBS (50%, *v*/*v*) at 37 °C for 24 h. The particle size of nanoparticles were determined at different time interval. After incubated, samples were mixed with excess heparin solution to replace pDNA in the acidic condition (pH 5.5) for 30 min and electrophoresed on 0.7% agarose gel. After electrophoresis, gel was stained by ethidium bromide (EB) (10 μg/mL) and pictures were obtained by a Tanon 2500R automatic digital gel image analysis system (Shanghai Tanon company, Shanghai, China).

### 2.7. In Vitro Cytotoxicity

4T1 mouse breast cancer cells and human hepatoma HepG2 cells were used. 4T1 and HepG2 cells were cultured in RPMI 1640 medium with 10% FBS at 37 °C in an atmosphere of 5% CO_2_ humidified atmosphere, respectively. The cells were maintained in 96-well plate at an initial density of 5 × 10^3^ cells per well. After 12 h attachment, FA-PEG-Pam/CaP/NDs, PEG-Pam/CaP/NDs, FA-PEG-Pam/CaP/pDNA, CaP/NDs, NDs and pDNA (The pDNA concentration of all preparations was 1.25 μg/mL) were added into the plates, respectively. 20 μL MTT (5 mg/mL) was added to each well after incubation for 24, 48 and 72 h. Then, the plates were further incubated for 4 h. Before 150 μL DMSO was added, the wells were shaken for 5 min, all of the culture medium was removed. The absorbance of each sample was evaluated at 490 nm.

### 2.8. Cellular Uptake 

To study the cellular uptake of nanoparticles, the plasmid DNA was labeled with YOYO-1 dye. HepG2 cells and 4T1 cells were seeded in 6-well plates (5 × 10^5^ cells per well) overnight to allow attachment, respectively. Then, the plates were cultured with pDNA, mPEG-Pam/CaP/NDs, FA-PEG-Pam/CaP/NDs, Lipofectamine/NDs and CaP/NDs, respectively. The cells were washed twice with precooled PBS, trypsinized and resuspended. Finally, the samples were detected by a BD flow cytometer (BD Biosciences, San Jose, CA, USA).

### 2.9. In Vitro Transfection

HepG2 and 4T1 cells were seeded into 6-well plates (1 × 105 cells per well) and incubated in RPMI 1640 medium overnight to allow attachment. After that, medium was displaced by serum-free medium prior to adding the 120 μL complexes containing 0.5 μg pDsRed-M-N1 plasmid. After an incubation for another 24 h, plates were washed with precooled PBS and treated with Hoechst 33258 (10 μg/mL, Ex: 345 nm; Em: 478 nm) for 0.5 h. The transfection of red fluorescence protein (RFP, Ex: 556 nm; Em: 586 nm) were determined by LSCM (Olympus, Tokyo, Japan). Then, cells were washed twice with precooled PBS, trypsinized and resuspended. Finally, samples were detected by BD flow cytometer. Transfection efficiency was defined as the percentage of RFP-positive cells.

### 2.10. Apoptosis Experiment

4T1 cells were seeded in a 6-well plate at a concentration of 1 × 10^6^ cells/well. Different samples were added into each well and incubation for 48 h. The following procedures was carried out according to the guideline of the AnnexinV-FITC/PI cell apoptosis kit and analyzed by flow cytometry. In addition, the content of Caspase-3, Caspase-8 and Caspase-9 solution was assessed by the Bradford method according to the kit instructions.

### 2.11. Biodistribution in Tumor-Bearing Mice 

Female BALB/c mice were injected with 4T1 cell suspension (5 × 10^6^ cells in 0.2 mL PBS) which was subcutaneously inoculated into the right axillary fossa of mice. When the tumor reached approximately 100 mm^3^, different formulations (0.2 mL) were injected via the tail vein at a dosage of 2.5 μg pDsRed-M-N1 plasmid per tumor-bearing mouse, respectively. The expression of RFP was assessed by IVIS image system (Carestream Health, Rochester, NY, USA) at different time point.

### 2.12. Tumor Suppression Efficiency

The antitumor effect of nanoparticles was evaluated on the 4T1 tumor xenograft mouse model. Meanwhile, tumor suppressor p53 gene was set as a therapeutic gene to investigate the tumor suppression efficiency in vivo. Mice were separated into 4 groups (*n* = 16): Saline, CaP/NDs, Lipofectamine/NDs and FA-PEG-Pam/CaP/NDs group. Mice were injected with 0.2 mL of formulation via the tail vein every 3 days for 4 times, while the dosage of p53 gene was 2 mg/kg. The tumor size and body weight of the mice were measured every 2 days. The tumor volume was calculated by the following formula:

Tumor volume = (length × width^2^)/2 (2)

At the 14th day, six mice were sacrificed and the other mice were left for survival investigation. The inhibition rate (IR%) was calculated according to the following equation:

IR (%) = (Ws − Wt)/Ws × 100%
(3)

Ws and Wt represent the tumor weight of control group and treatment group, respectively. Then, p53 protein expression in tumors was analyzed by Western Blotting. In addition, H&E assay was performed. All animal experiments were pursuant to the rules of Experimental Animal Administrative Committee of Shenyang Pharmaceutical University and the committee approved the experiments (SYPU-IACUC-C2019-5-15-105, 15 May 2019).

### 2.13. Statistics 

All the experiments were repeated at least three times. Results are presented as mean ± standard deviation (SD). Pairwise comparisons between treatments were made using Student’s t-test. A *p*-value < 0.05 was considered statistically significant.

## 3. Results and Discussion

### 3.1. Synthesis and Characteristics of FA-PEG-Pam

In the ^1^H-NMR spectrum (Figures S2, S3 and S4), the following characteristic peaks of FA-PEG-Pam were observed: FA-PEG-COOH: δ_a_ 8.63 ppm (–N=CH–C–), δ_b_ 3.85 ppm (–C–CH_2_–NH–), δ_C_ 6.63 ppm (–C–CH=C–), δ_d_ 7.71 ppm (–C=CH–C–), δ_e_ 1.26 ppm (–CH(COOH)–CH_2_–C–) and δ_f_ 3.63 ppm(–CH_2_–CH_2_–O–); Pam: δ_g_ 3.24 ppm (–NH–CH_2_–C–) and δ_h_ 1.88 ppm (–C–CH_2_–C–)_._ These characteristic peaks indicated the pamidronate was successfully conjugated to FA-PEG-COOH chains. Meanwhile, the FTIR data (Figures S5, S6 and S7) of FA-PEG-Pam exhibited several bands at 3424.9 cm^−1^ (υ_O–H_ in Pam and FA), 2888.0 cm^−1^ (υ_C–H_ in PEG), 1488.1 cm^−1^ (υ_C=C_ in FA), and 1110.2 cm^−1^ (υ_C–O–C_ in PEG). From the results above, we can conclude that FA-PEG-Pam was successfully prepared.

### 3.2. Characterization of FA-PEG-Pam/CaP/NDs Nanoparticles

According to a previous report, the preparation of CaP nanoparticles in aqueous solution will lead to the formation of insoluble large aggregates over time [21]. The large size and instability of CaP/DNA nanoparticles hinders their use for in vivo delivery [22,23,24,25,26,27,28]. Therefore, the FA-PEG-Pam shell was employed to coat CaP nanoparticles for enhancing the colloidal stability via its hydrophilic effect. As shown in Table 1, the size of FA-PEG-Pam/CaP/NDs nanoparticles was 164.2 ± 7.6 nm, which was smaller than CaP/NDs.

The particle size of CaP/NDs further increased at 24 h after preparation (Table 2). This demonstrated that FA-PEG-Pam could prevent particles from aggregation.

**Figure 2:**
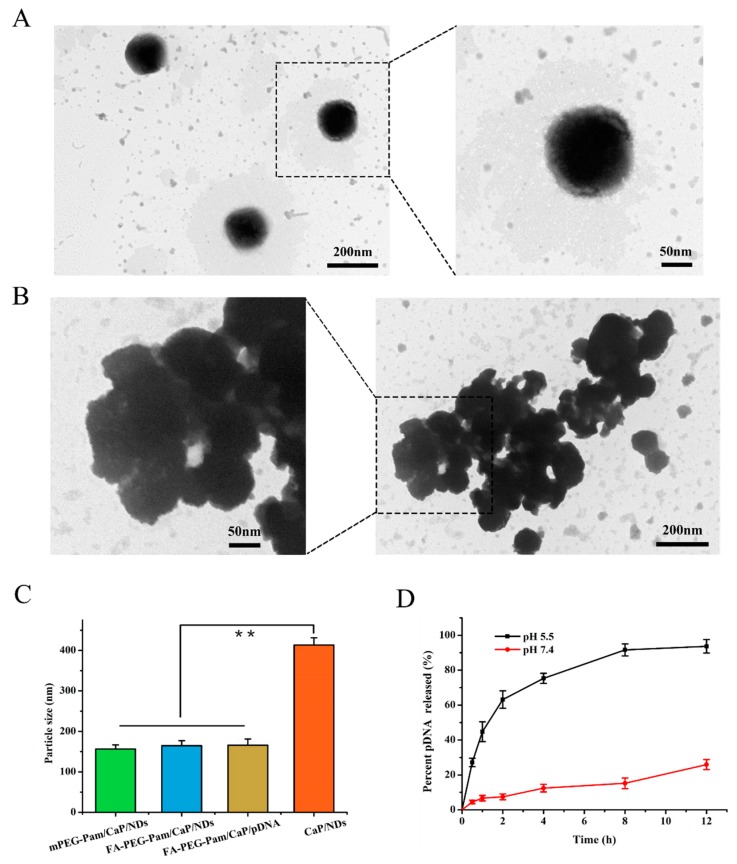
Transmission electron micrographs of (**A**) FA-PEG-Pam/CaP/NDs, and (**B**) CaP/NDs nanoparticles. (**C**) Comparison of the size of mPEG-Pam/CaP/NDs, FA-PEG-Pam/CaP/NDs, FA-PEG-Pam/CaP/pDNA and CaP/NDs nanoparticles. Samples were freshly prepared and diluted ten times with deionized water. (**D**) In vitro release of pDNA from FA-PEG-Pam/CaP/NDs nanoparticles at different pH value. Data presented as mean ± SD (*n* = 3), ** *p* < 0.01.

Besides, the zeta potential of FA-PEG-Pam/CaP/NDs remained essentially neutral (−1.23 ± 0.14 mV). Furthermore, there was no significant difference between FA-PEG-Pam/CaP/pDNA and FA-PEG-Pam/CaP/NDs (*p* > 0.05, Figure 2C), which indicated the NLS peptide had no obvious effect on particle size. Meanwhile, the morphology of the nanoparticles was observed by TEM. There was an obvious aggregation in CaP/NDs nanoparticles (Figure 2B), while nanoparticles with FA-PEG-Pam were well dispersed (Figure 2A). This phenomenon was mainly due to the fact that pamidronate could steadily interact with CaP nanoparticles through the so-called “bone hook” hydroxyl group and its two phosphonate groups, thereby inhibiting the aggregation of calcium CaP/DNA [29,30].

### 3.3. Stability Analysis of Nanoparticles

**Figure 3:**
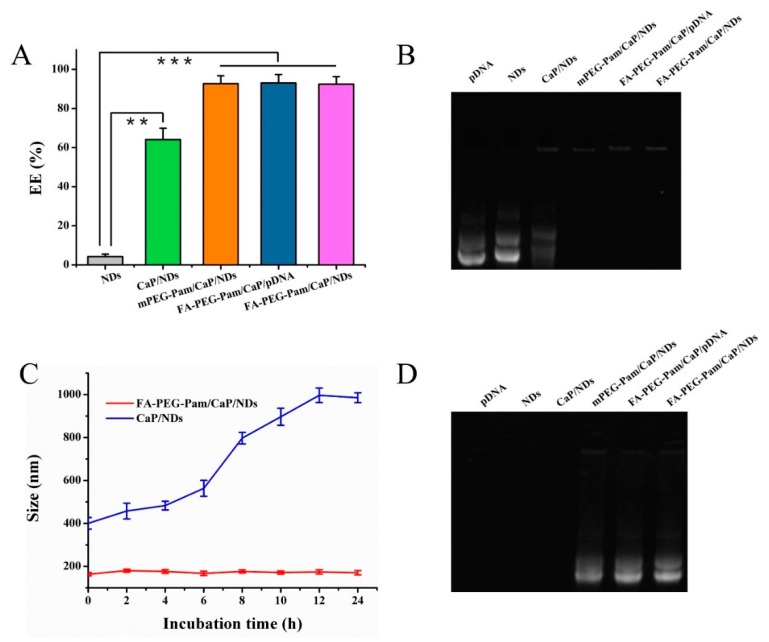
Encapsulation efficiency of different formulations by (**A**) Hoechst 33258 intercalation, and (**B**) gel retardation assay. (**C**) Changes in particle size of FA-PEG-Pam/CaP/NDs and CaP/NDs nanoparticles following incubation at fetal bovine serum. Data are shown as mean ± SD (*n* = 3). (**D**) In vitro serum stability assay of FA-PEG-Pam/CaP/NDs and its reference formulations by agarose gel electrophoresis. ** *p* < 0.01, and *** *p* < 0.001.

The pDNA encapsulation efficiency (EE%) was evaluated by a gel retardation assay and Hoechst 33258 intercalation. As shown in Figure 3A, the EE% of mPEG-Pam/CaP, FA-PEG-Pam/CaP/pDNA and FA-PEG-Pam/CaP/NDs were all above 90% and there was no obvious difference among them (*p* > 0.05). The result was consistent with the gel retardation assay (Figure 3B).

It is generally known that CaP nanoparticles frequently display poor reproducibility and low in vivo transfection efficiency because of their uncontrollable aggregation in serum [31]. Thus, in vitro serum stability was evaluated via observing the change of particle size. The size of FA-PEG-Pam/CaP/NDs was relatively stable, with no distinct changes, even after 24 h (Figure 3C). In comparison, the particle size of CaP/NDs further increased. The results above indicated that FA-PEG-Pam could protect CaP/NDs nanoparticles from aggregation. In order to further investigate whether our preparations owned desired serum stability, after incubated with FBS, samples were mixed with excess heparin solution to replace pDNA in the acidic condition (pH 5.5) for 30 min and electrophoresed. The strip of FA-PEG-Pam/CaP/NDs nanoparticles was still clear, while NDs, free pDNA and CaP/NDs had no obvious bands (Figure 3D), suggesting the nanoparticles with FA-PEG-Pam shell could protected DNA against degradation in serum to some extent.

### 3.4. pH-Sensitive Release of pDNA from FA-PEG-Pam/CaP/NDs Nanoparticles

Endosomal entrapment has been regarded as the uppermost intracellular block for pDNA delivery [32]. The pDNA entrapped in the endosome would be decomposed by the highly acidic environment, resulting in an extremely low transfection efficiency [33,34]. It was expected that FA-PEG-Pam/CaP/NDs nanoparticles could dissolve in the weakly acidic solution. The released Ca^2+^ ion could increase the osmotic pressure, and the embedded pDNA could escape the endosome by endosome rupture owing to the increase of osmotic pressure [35]. Hence, the release of pDNA from the nanoparticles at the endosome (pH 5.5) and physiological environment (pH 7.4) were evaluated. As illustrated in Figure 2D, less than 20% of the pDNA was released at neutral pH even after 12 h. In contrast, there are approximately 45% of pDNA released after 1 h and nearly 75% released after 4 h at pH 5.5. From the abovementioned results, we can draw the conclusion that FA-PEG-Pam/CaP/NDs nanoparticles could be stable in the physiological environment, and dissociate in a weakly acidic solution.

### 3.5. MTT Assay

**Figure 4:**
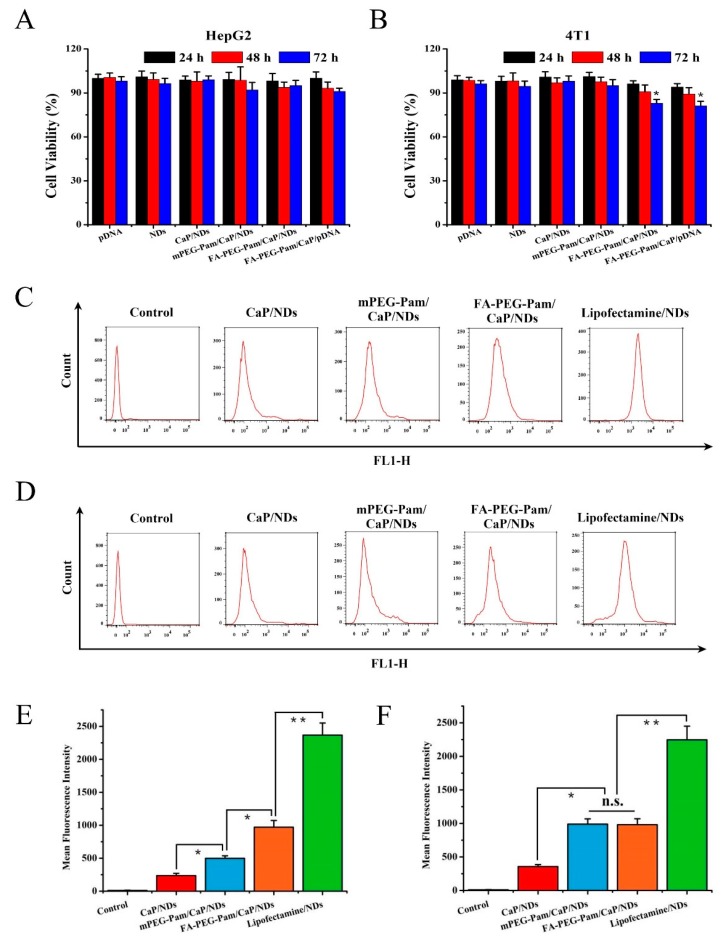
In vitro cytotoxicity of FA-PEG-Pam/CaP/NDs and its reference formulations against (**A**) HepG2 and (**B**) 4T1 cells by MTT assay. Flow cytometry measurement of the cellular uptake of different formulations against 4T1 cells (**C**,**E**) and HepG2 cells (**D**,**F**). * *p* < 0.05 and ** *p* < 0.01.

Folate receptor is overexpressed in 4T1 cells, but rarely expressed in HepG2 cells, so 4T1 cells and HepG2 cells were selected for experiment [36,37,38,39]. Herein, cytotoxicity of FA-PEG-Pam/CaP/NDs and its reference formulations was evaluated on HepG2 and 4T1 cells by the MTT assay. As exhibited in Figure 4A, FA-PEG-Pam/CaP/NDs showed no remarkable cytotoxicity in HepG2 cells at 72 h (*p* > 0.05). In contrast, an obvious difference (*p* < 0.05) was found in 4T1 cells (Figure 4B), implying that FA-PEG-Pam/CaP/NDs had more cytotoxic to 4T1 cells at the same time point. Similarly, no significant difference was found between each preparation against HepG2 cells at 72 h (*p* > 0.05), while FA-PEG-Pam/CaP/NDs were apparently more cytotoxic than mPEG-Pam/CaP/NDs against 4T1 cells. It is worth noting that FA-PEG-Pam/CaP/NDs exhibited more cytotoxicity than reference preparations against 4T1 cells at all time points. This increased cytotoxicity could be ascribed to the folate receptors (FR)-mediated endocytosis [40]. But the cell viability was still over 80% even if treated with FA-PEG-Pam/CaP/NDs for 72 h, implying FA-PEG-Pam/CaP/NDs is a low cytotoxicity gene delivery system.

### 3.6. Cell Uptake Study

The mean fluorescence intensity of various formulations was measured by flow cytometry. As illustrated in Figure 4C,E, FA-PEG-Pam/CaP/NDs showed stronger fluorescent signal than mPEG-Pam/CaP/NDs in FR-positive 4T1 cells (*p* < 0.05), indicating FA-mediated targeting enhanced the cellular uptake of nanoparticles. In contrast, there was no significant difference between FA-PEG-Pam/CaP/NDs and mPEG-Pam/CaP/NDs in FR-negative HepG2 cells (*p* > 0.05) (Figure 4D,F). Therefore, it could be speculated that the FA segment could promote the cellular uptake of FA-PEG-Pam/CaP/NDs via the FA receptor modified endocytosis. In addition, mPEG-Pam/CaP/NDs had better cellular uptake than CaP/NDs. This phenomenon should be due to the higher particle size of CaP/NDs, which was not conducive to the endocytosis.

### 3.7. In Vitro Transfection and Expression

**Figure 5:**
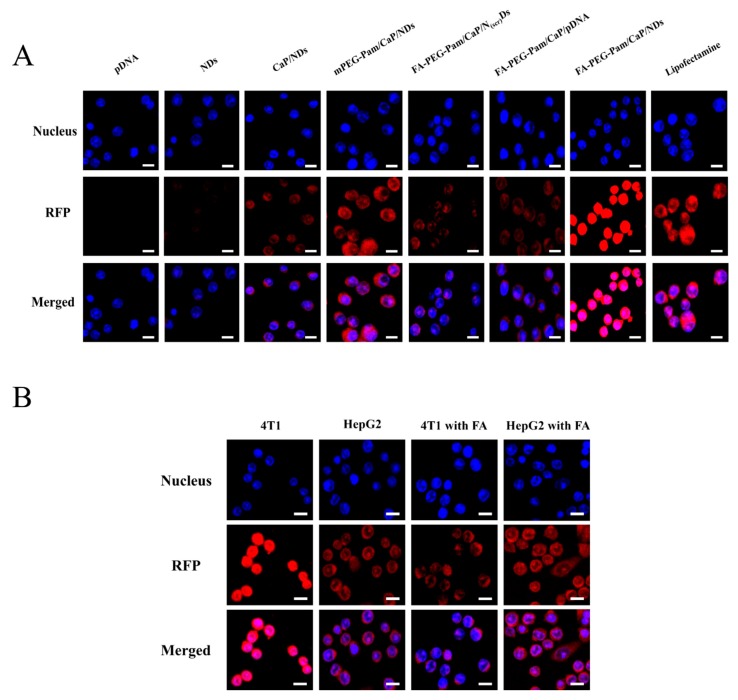
(**A**) In vitro transfection and expression of RFP was analyzed by LSCM in 4T1 cells. (**B**) Targeting ability of FA-PEG-Pam/CaP/NDs and competitive inhibition of free folic acid. Fluorescence intensity of RFP expressed in HepG2 and 4T1 cells treated with FA-PEG-Pam/CaP/NDs, with or without the presence of free folic acid. Cells were treated with 1 mM folic acid medium or folic acid-free medium before incubating with FA-PEG-Pam/CaP/NDs. Scale bar in all pictures indicates 40 µm.

**Figure 6:**
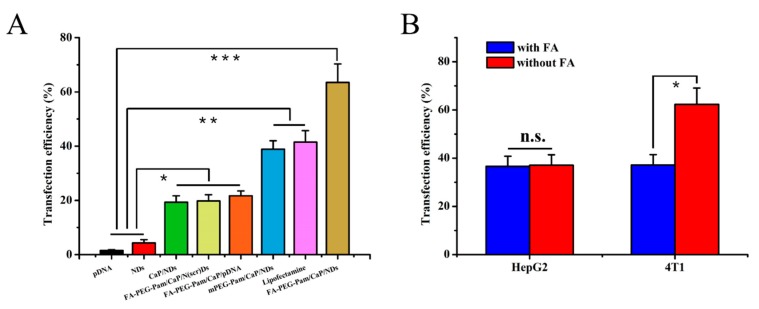
Transfection efficiency was defined as the percentage of RFP-positive cells and determined by flow cytometry. Transfection efficiency of RFP in (**A**) 4T1 cells and (**B**) HepG2 and 4T1 cells treated with FA-PEG-Pam/CaP/NDs, with or without the presence of free folic acid. * *p* < 0.05, ** *p* < 0.01, and *** *p* < 0.001.

Besides cytotoxicity, the transfection efficiency of nanoparticles is another crucial factor to be considered during the design of gene carriers. It is widely confirmed that the benign transfection of nanoparticles is obligatory for gene delivery. Herein, the transfection efficiency of nanoparticles was assessed using pDsRed-M-N1 plasmid as a reporter gene. As illustrated in Figure 5A and Figure 6A, compared with FA-PEG-Pam/CaP/pDNA (21.7%), FA-PEG-Pam/CaP/NDs exhibited a greater gene transfection level (63.5%, *p* < 0.05). A viable explanation of this phenomenon is the presence of NLS peptides. As far as we know, NLS peptides have been widely applied for gene delivery due to its capability of improving the nuclear transfer for DNA [41]. To further affirm the role of NLS peptides upon transfection, we set a scrambled sequence NLS_(scr)_ to form the FA-PEG-Pam/CaP/NLS_(scr)_/pDNA (shorten as FA-PEG-Pam/CaP/N_(scr)_Ds) as control. It is clear that FA-PEG-Pam/CaP/NDs (63.5%) showed a higher transfection efficiency than FA-PEG-Pam/CaP/N_(scr)_Ds (19.8%), which stressed the importance of NLS peptide again. In addition, FA-PEG-Pam/CaP/NDs exhibited better transfection efficiency than mPEG-Pam/CaP/NDs (*p* < 0.05) before our eyes. This result would be ascribed to the existence of the FA moiety, which possessed valid combination with FR-positive 4T1 cells. Meanwhile, FA-PEG-Pam/CaP/NDs showed preferable transfection than positive control group (Lipofectamine 2000, *p* < 0.05), which is not consistent with the cellular uptake. Maybe the endosomal entrapment could be a proper explanation for this interesting phenomenon. The results above revealed that FA-PEG-Pam/CaP/NDs possessed favorable transfection efficiency.

To ascertain the tumor target ability of prepared nanoparticles, HepG2 and 4T1 cells were treated with FA-PEG-Pam/CaP/NDs in medium with or without free FA, respectively. Relatively higher transfection efficiency of FA-PEG-Pam/CaP/NDs was achieved in 4T1 cells (*p* < 0.05) (Figure 5B and Figure 6B). When treated with 1 mM FA, the fluorescence apparently weakened and there was no clear difference between 4T1 and HepG2 cells (*p* > 0.05). The interesting phenomenon demonstrated FA-PEG-Pam/CaP/NDs showed higher expression in FR-positive 4T1 cells (62.3%) than that in FR-negative HepG2 cells (37.1%).

### 3.8. Apoptosis Experiment

**Figure 7:**
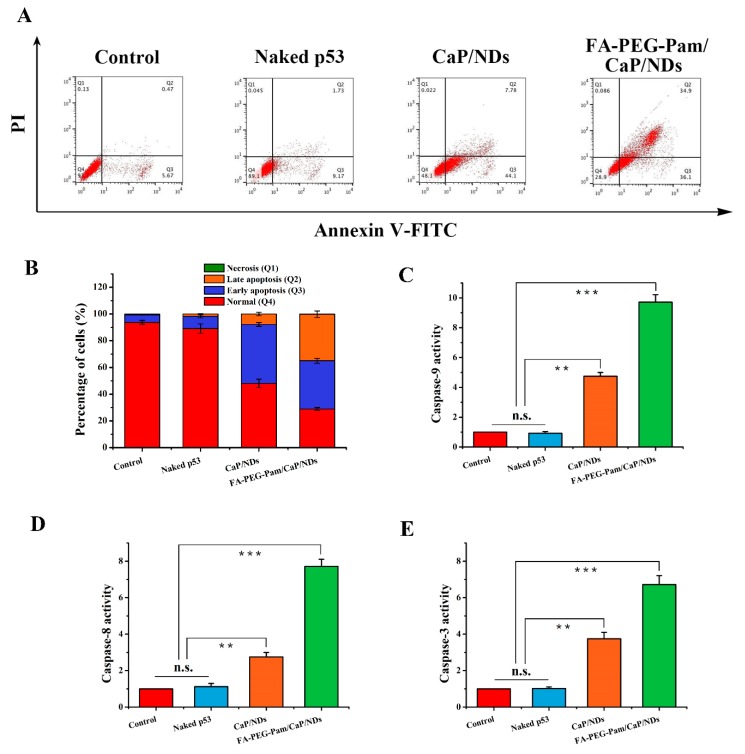
(**A**) and (**B**) apoptosis-inducing effect of FA-PEG-Pam/CaP/NDs and its reference formulations. Determination of (**C**) Caspase-9, (**D**) Caspase-8 and (**E**) Caspase-3 activity. ** *p* < 0.01, and *** *p* < 0.001.

Herein, we utilized p53 plasmid as a therapeutic gene to carry out the apoptosis experiment. In recent years, two caspase apoptosis signaling pathways have been identified. One of them is the mitochondria-mediated internal apoptotic pathway [42,43]. Briefly, the production of reactive oxygen species would lead to the decrease of transmembrane potential, which makes the mitochondrial membrane gap release the cytochrome c and apoptosis-inducing factor (AIF) [44]. The combination between cytochrome c and apoptosis protease activating factor-1 (Apaf-1) will activate the caspase-9. Afterwards, the caspase-3 will be activated, which will ultimately activate the Ca^2+^–Mg^2+^ dependent endonuclease, thereby initiating apoptosis. Another one is the death receptor-mediated external apoptotic pathway [45,46]. The cells are stimulated by apoptosis signals and the death receptors such as tumor necrosis factor (TNF) are activated, which further makes the caspase-8 and caspase-3 initiate cell death [47]. Meanwhile, the caspase-8 has a certain positive activation effect on the caspase-9. It can be easily found that both pathways will eventually activate caspase-3, which is the executor of apoptosis. As shown in Figure 7C–E, compared with the control group, the content of three caspase enzyme in FA-PEG-Pam/CaP/NDs group was significantly increased, indicating that the apoptosis pathway was activated after administration. Notably, the enzyme activity of FA-PEG-Pam/CaP/NDs group was stronger than CaP/NDs group. The results of the three enzyme activities indicate that p53 can simultaneously activate two caspase-dependent apoptotic signaling pathways. In addition, annexin V-FITC/PI double staining is an effective mean in the detection of apoptosis by flow cytometry. As shown in Figure 7A,B, FA-PEG-Pam/CaP/NDs nanoparticles possessed the strongest apoptosis (71.1%, including early apoptosis, late apoptosis, and necrosis cell), indicating that they had a desired effect on inducing apoptosis, which is consistent with the detection of caspase enzyme activity.

### 3.9. Biodistribution and Tumor Suppression Efficiency of Different Nanoparticles

**Figure 8:**
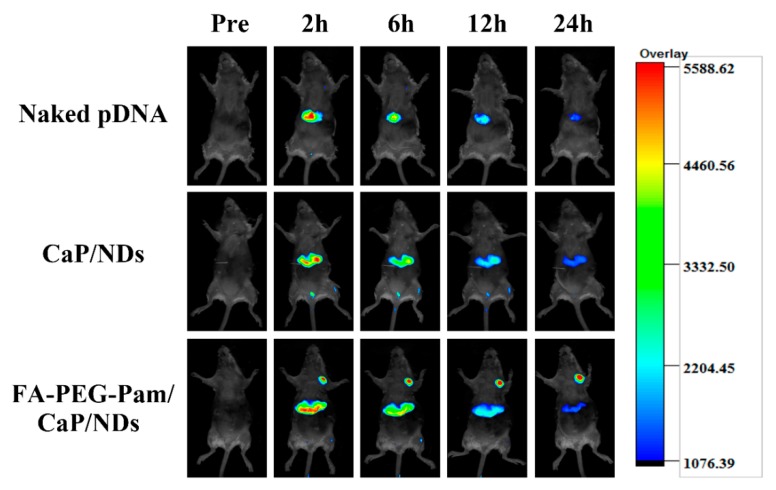
In vivo image of RFP gene expression delivered by FA-PEG-Pam/CaP/NDs and its reference formulations injected into bearing 4T1 tumor xenografts BALB/c mice monitored by an NIR fluorescence imaging system.

From the in vitro assays above, we found FA-PEG-Pam/CaP/NDs had favorable tumor target ability and transfection efficiency. To ascertain whether these features would be realized in vivo, the expression of red fluorescence protein (RFP) in tumor-bearing mice was investigated using a NIR fluorescence imaging system. Figure 8 shows the images of samples treated with various formulations in 4T1 tumor-bearing mice, respectively. Naked pDNA was mainly distributed in liver and was generally metabolized. CaP/NDs presented similar biodistribution behavior to naked pDNA. This result could be attributed to the relative instability of CaP/NDs in vivo, they may aggregate and be cleared in circulation. In contrast, FA-PEG-Pam/CaP/NDs displayed favorable accumulation at tumor issue and the fluorescence sustained for 24 h. This phenomenon suggested the excellent tumor target ability and desirable feature for long-circulating in vivo.

**Figure 9:**
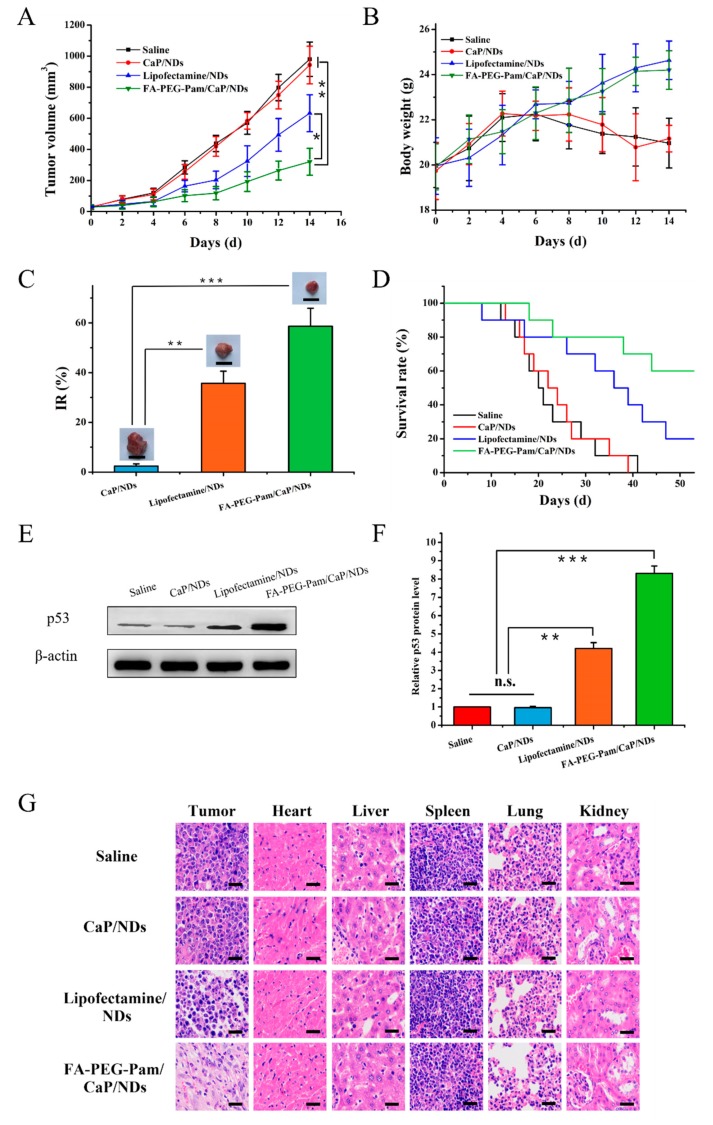
In vivo antitumor activity. The mean (**A**) tumor volume, (**B**) body weight, (**C**) inhibition rate and tumor graph. Scale bar indicates 1 cm. (**D**) survival rate analysis of BALB/c mice bearing 4T1 tumor xenografts, after intravenous administration of Saline, CaP/NDs, Lipofectamine/NDs and FA-PEG-Pam/CaP/NDs. (**E**) and (**F**) Western blotting analysis of p53 protein expression in tumor xenografts transfected with different formulations. (**G**) H&E staining of tumors of different groups. Blue and pink represent nucleic and cytoplasm, respectively. Red circle indicated apoptosis/necrosis regions and scale bars represent 100 μm. * *p* < 0.05, ** *p* < 0.01, and *** *p* < 0.001.

To assess the antitumor efficacy in vivo, female BALB/c mice bearing 4T1 tumor xenografts were used. As far as we know, tumor suppressor gene p53 is able to regulate the normal cell growth in DNA replication, while its signal transduction pathway acts a predominant part in cell growth, senescence and malignancy [48]. When p53 gene is affected by some undesirable factors, like excessive UV radiation, it will mutate. Mutant p53 not only loses its original tumor suppressing function, but also results in tumorigenesis and invasiveness in vivo [49]. In the case, we utilized p53 gene as a therapeutic gene to perform the antitumor experiment in vivo. As shown in Figure 9A, all groups exhibited no remarkable difference in tumor volume in the first few days, while treatment groups showed growth inhibition after 4 days. We measured and weighed tumors, then calculated the inhibition rate (IR%). There was no obvious difference between saline group and CaP/NDs (Figure 9C). Most probably, CaP/NDs lacks the capacity for long-circulating, which leads to the aggregation of nanoparticles in injected region and increased eliminate rate, thus had an indistinctive effect on tumors. Lipofectamine group showed moderate antitumor efficiency (IR% = 35.7%). Hopefully, FA-PEG-Pam/CaP/NDs displayed a highest inhibition rate (IR% = 58.7%), this could be attributed to the presence of the FA-PEG-Pam shell, which indicated the preferable tumor suppression efficiency of nanoparticles, arising from the proper particle size, desired stability, tumor target ability and long-circulating. On the other hand, the changes in body weight are considered as a direction for safety. As Figure 9B showed, the body weight of treatment groups was heavier than the saline group, suggesting the preferable compatibility of nanoparticles. Meanwhile, as shown in Figure 9D, the survival rate of FA-PEG-Pam/CaP/NDs was higher than any other group, further suggesting FA-PEG-Pam/CaP/NDs was a suitable gene delivery system.

To further prove the tumor growth inhibition was attributed to p53 plasmid, p53 protein expression in tumors was detected by western blotting. As shown in Figure 9E,F, there was an obvious upregulation in the p53 protein expression level. FA-PEG-Pam/CaP/NDs group was 8.3-fold higher than the saline group. Furthermore, H&E staining was used to analysis the histological changes. Figure 9G illustrates that FA-PEG-Pam/CaP/NDs had the largest apoptosis/necrosis region in comparison with other formulations. Meanwhile, no evident toxicity to major organs was found.

## 4. Conclusions

In this paper, a multifunctional gene delivery system, FA-PEG-Pam/CaP/NDs nanoparticles, has been successfully constructed. The FA-PEG-Pam shell was employed to coat CaP nanoparticles for enhancing the colloidal stability via its hydrophilic effect. Compared with CaP/NDs (413.5 ± 17.9 nm), FA-PEG-Pam/CaP/NDs exhibited smaller particles size (164.2 ± 7.6 nm) and favorable serum stability. pDNA from the nanoparticles released fast in the endosome (75% after 4 h) and hardly released in the physiological environment (less than 20% after 12 h). FA-PEG-Pam/CaP/NDs had better cellular uptake than mPEG-Pam/CaP/NDs in FR-positive 4T1 cells, which could be attributed to the high-affinity interactions between FA and highly expressed FR. FA-PEG-Pam/CaP/NDs had low cytotoxicity and desired effect on inducing apoptosis (71.1%). Furthermore, FA-PEG-Pam/CaP/NDs exhibited admirable transfection efficiency (63.5%) with the help of NLS. Meanwhile, FA-PEG-Pam/CaP/NDs showed higher expression in FR-positive 4T1 cells than that in FR-negative HepG2 cells. More importantly, in vivo evaluation confirmed that FA-PEG-Pam/CaP/NDs they possessed the highest tumor growth inhibition (IR% = 58.7%) among all preparations. Overall, the desired targeting ability and transfection efficiency in vitro and in vivo imply that this effective delivery system is a suitable strategy for gene therapy.

## Figures and Tables

**Table 1:** The particle size and zeta potential of nanoparticles.

	Particle Size (nm)	PDI	Zeta Potential (mV)
mPEG-Pam/CaP/NDs	156.4 ± 10.2	0.23 ± 0.05	−2.33 ± 0.28
FA-PEG-Pam/CaP/NDs	164.2 ± 7.6	0.17 ± 0.04	−1.23 ± 0.14
FA-PEG-Pam/CaP/pDNA	166.2 ± 15.2	0.15 ± 0.06	−0.83 ± 0.26
CaP/NDs	413.5 ± 17.9	0.26 ± 0.08	2.73 ± 1.13

**Table 2:** The particle size and zeta potential of nanoparticles at 24 h after preparation.

	Particle Size (nm)	PDI	Zeta Potential (mV)
mPEG-Pam/CaP/NDs	161.4 ± 8.3	0.21 ± 0.06	−1.47 ± 0.32
FA-PEG-Pam/CaP/NDs	169.0 ± 9.7	0.14 ± 0.05	−1.76 ± 0.21
FA-PEG-Pam/CaP/pDNA	164.2 ± 12.6	0.20 ± 0.07	0.43 ± 0.15
CaP/NDs	913.5 ± 56.9	0.27 ± 0.07	3.68 ± 1.45

## Supplementary Materials

The following are available online at https://www.mdpi.com/1999-4923/11/9/468/s1, Figure S1: The synthesis procedures of FA-PEG-Pam, Figure S2: The 1H-NMR spectrum of FA-PEG-COOH, Figure S3: The 1H NMR spectrum of Pam, Figure S4: The 1H NMR spectrum of FA-PEG-Pam, Figure S5: The FTIR spectrum of FA-PEC-COOH, Figure S6: The FTIR spectrum of Pam, Figure S7: The FTIR spectrum of FA-PEG-Pam.
