# Supplementary material for: A Valid Bisphosphonate Modified Calcium Phosphate-Based Gene Delivery System: Increased Stability and Enhanced Transfection Efficiency In Vitro and In Vivo
Source: Pharmaceutics. 2019 Sep 11;11(9):468. doi: 10.3390/pharmaceutics11090468 (PMC6781291; doi:10.3390/pharmaceutics11090468)
Supplement: Supplementary file 1 [file pharmaceutics-11-00468-s001.pdf]

pharmaceutics

MDPI

Supplementary Materials: A valid Bisphosphonate Modified Calcium Phosphate-Based Gene Delivery System: Increased Stability and Enhanced Transfection Efficiency In Vitro and In Vivo

Ming Zhao, Ji Li, Dawei Chen and Haiyang Hu

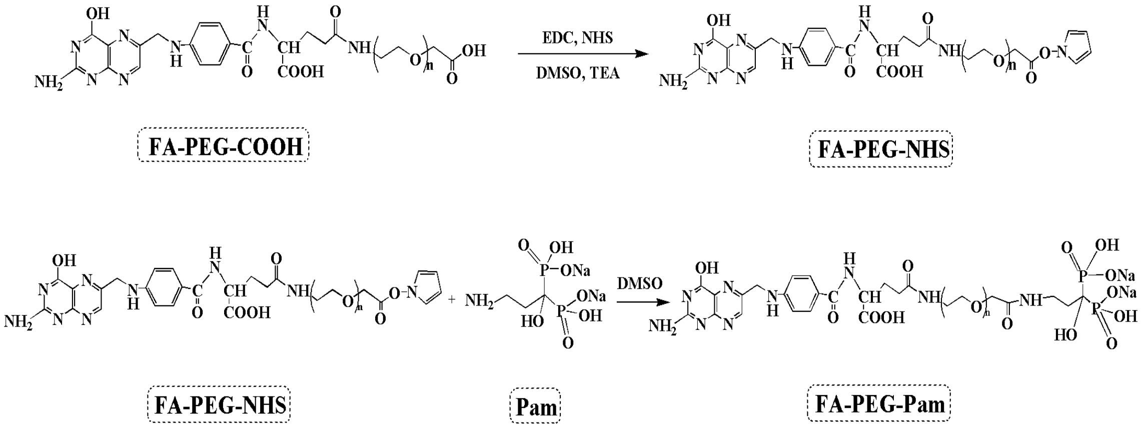

Figure S1. The synthesis procedures of FA-PEG-Pam.

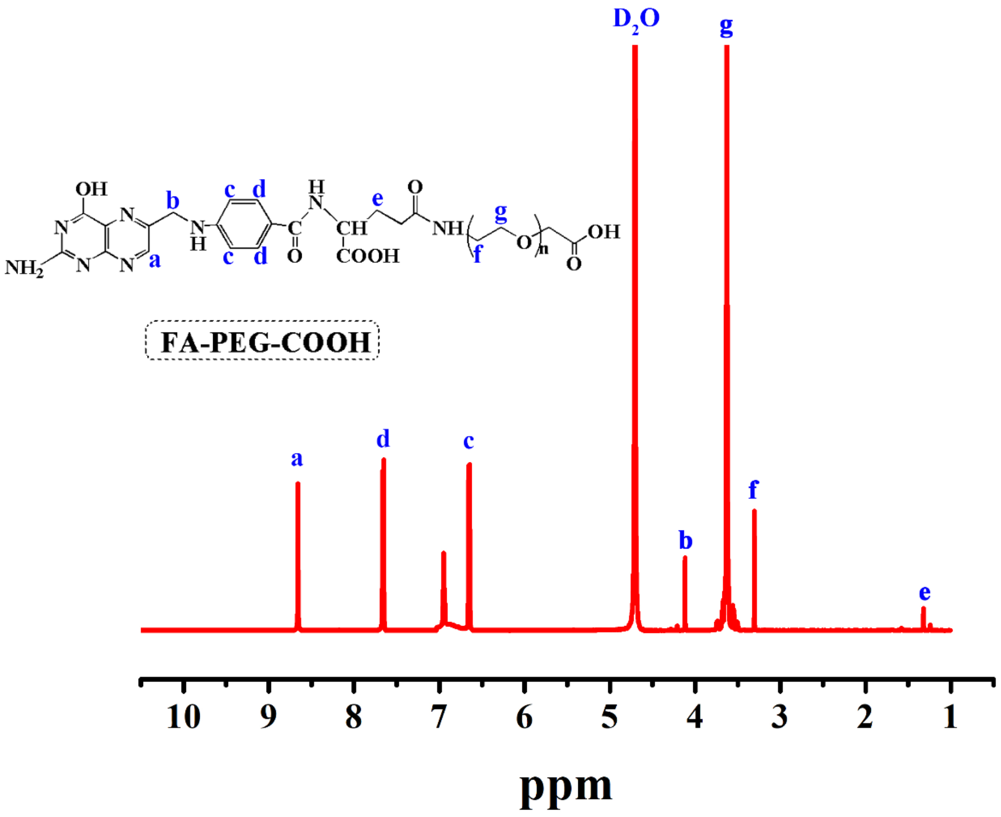

Figure S2. The ^{1}H NMR spectrum of FA-PEG-COOH.

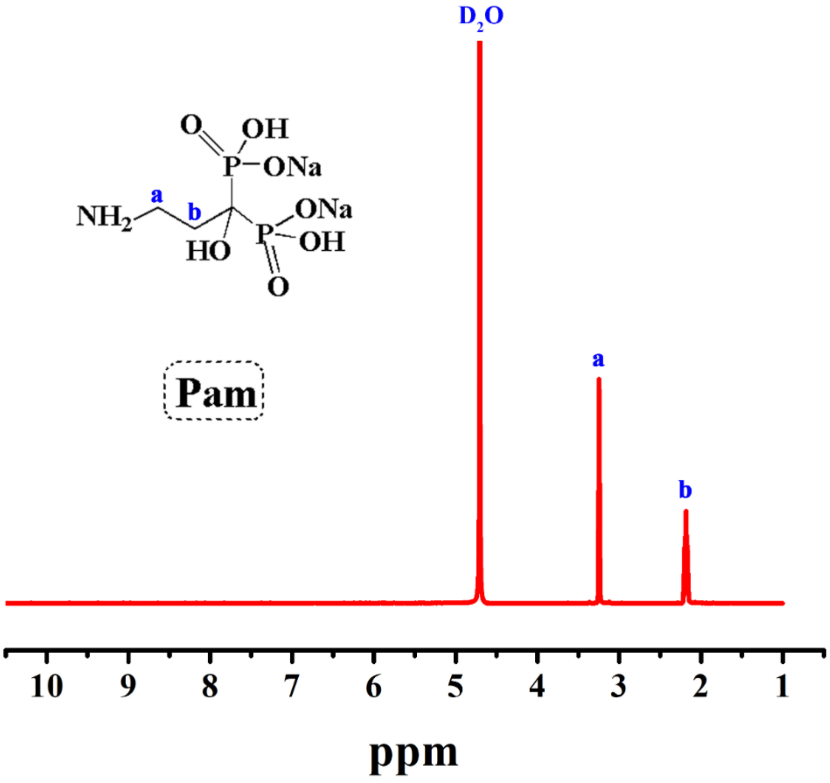

Figure S3. The ^{1}H NMR spectrum of Pam.

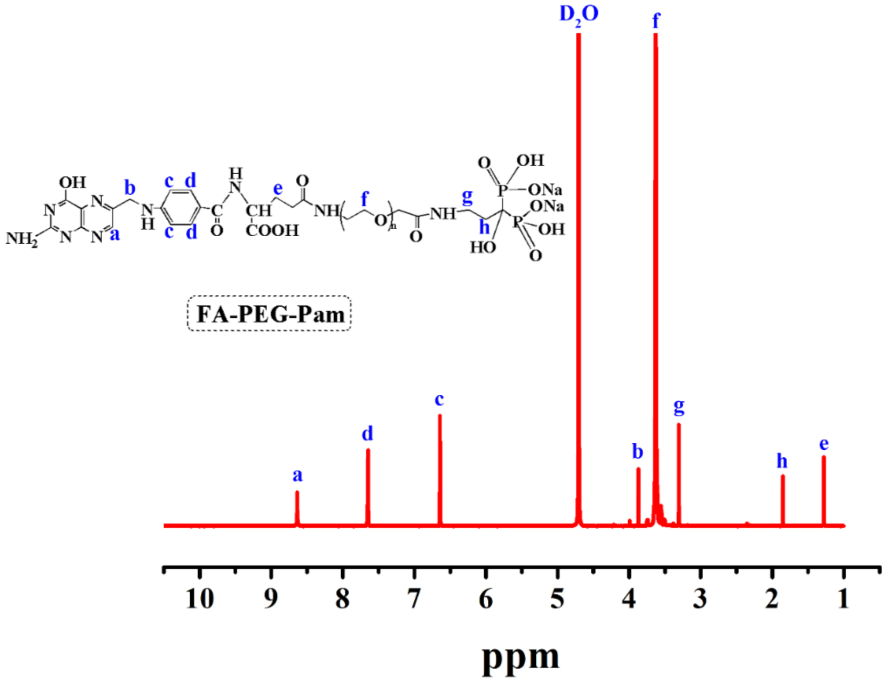

Figure S4. The ^{1}H NMR spectrum of FA-PEG-Pam.

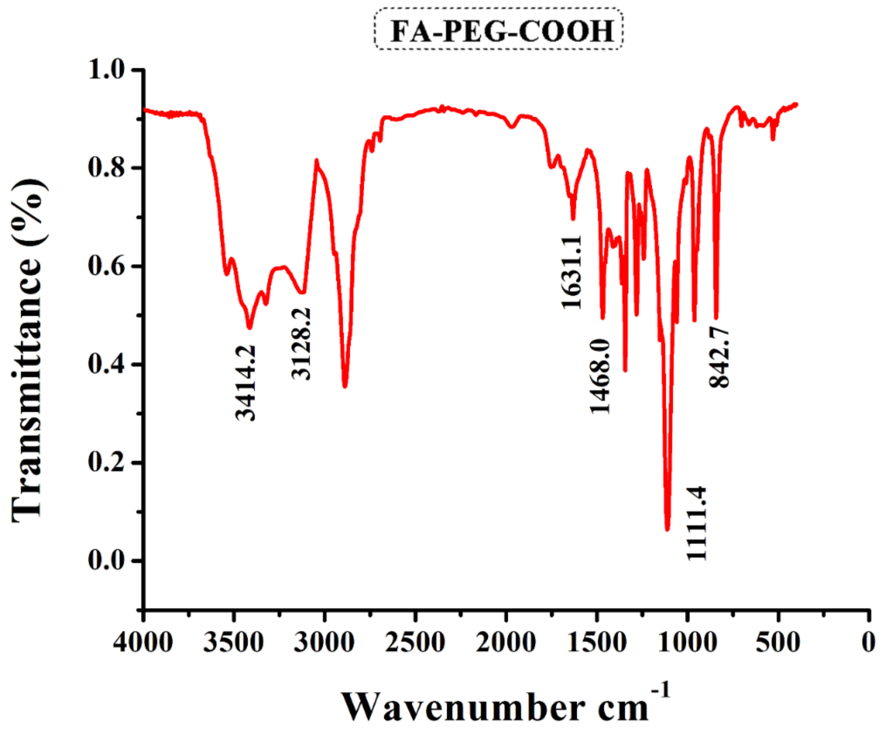

Figure S5. The FTIR spectrum of FA-PEC-COOH.

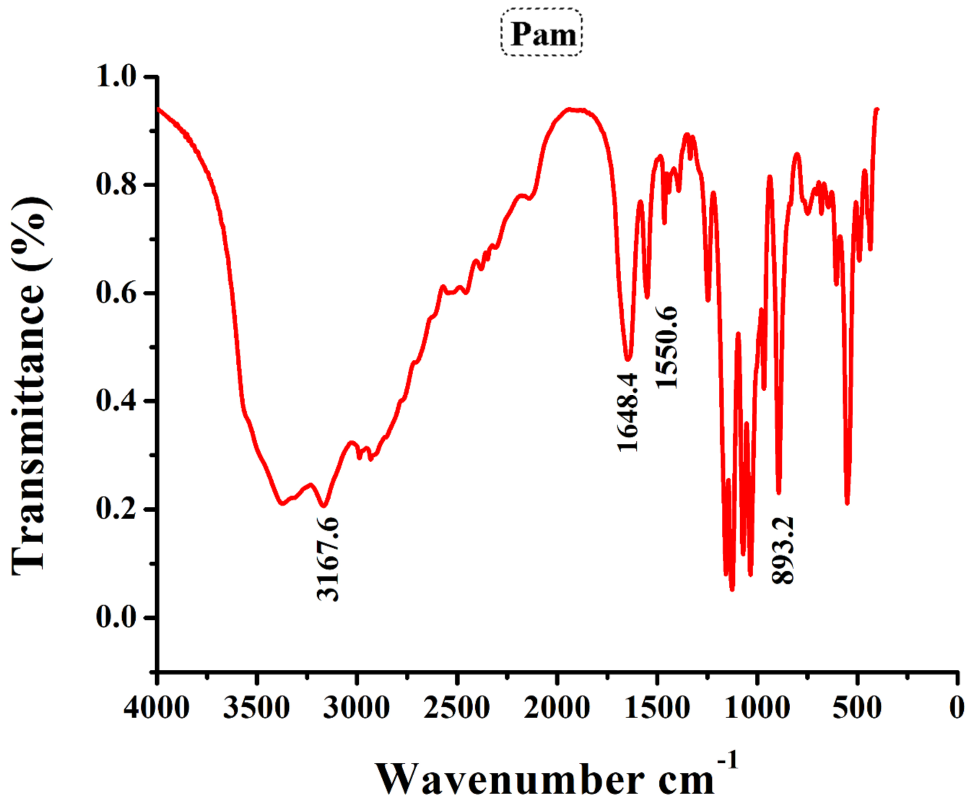

Figure S6. The FTIR spectrum of Pam.

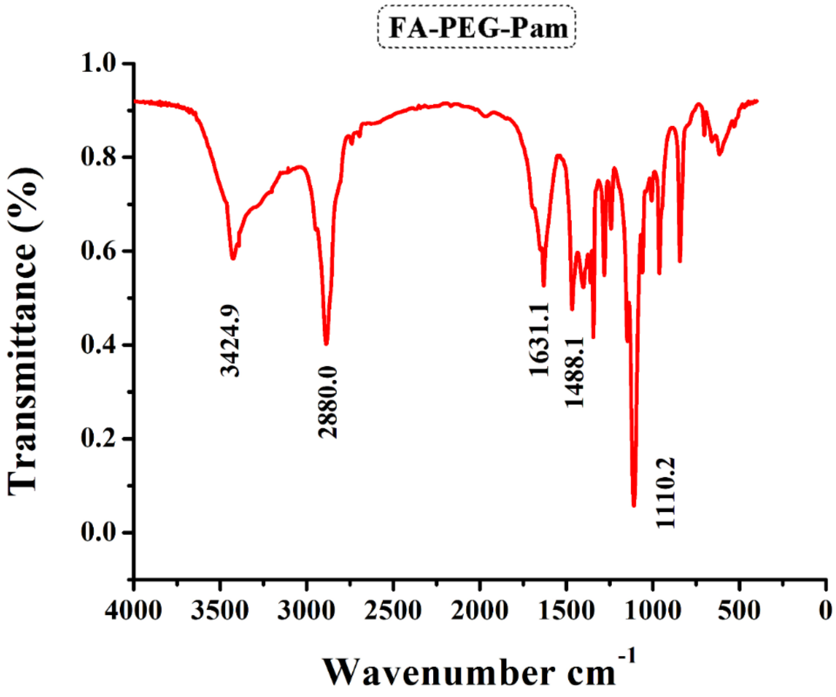

Figure S7. The FTIR spectrum of FA-PEG-Pam.
